# Hardware Impaired Self-Energized Bidirectional Sensor Networks over Complex Fading Channels †

**DOI:** 10.3390/s20195574

**Published:** 2020-09-29

**Authors:** Stefan R. Panic, Dushantha Nalin K. Jayakody, Sofiene Affes, Palanivelu Muthuchidambaranathan

**Affiliations:** 1 School of Computer Science and Robotics, National Research Tomsk Polytechnic University, 634050 Tomsk, Russia; 2 Faculty of Natural Science and Mathematics, University of Pristina, 38220 Kosovska Mitrovica, Serbia; 3 Centre for Telecommunication Research, Faculty of Engineering, Sri Lanka Technological Campus, Padukka 11500, Sri Lanka; 4 Institut National de la Recherche Scientifique Centre Énergie, Matériaux et Telecommunications, Montreal, QC H5A 1K6, Canada; affes@emt.inrs.ca; 5 ECE Department, National Institute of Technology Tiruchirappalli, Tamilnadu 620015, India; muthuc@nitt.edu

**Keywords:** hardware impairment, half-duplex, Hoyt fading channels, relay networks, Rician-shadowed fading channels, time-switching, wireless energy harvesting

## Abstract

Rapid emergence of wireless sensor networks (WSN) faces significant challenges due to limited battery life capacity of composing sensor nodes. It is substantial to construct efficient techniques to prolong the battery life of the connected sensors in order to derive their full potential in the future Internet of Things (IoT) paradigm. For that purpose, different energy harvesting (EH) schemes are relying on a wide array of sources. Following the same objective, in this work, we have observed a time-switching EH for half-duplex (HD) bidirectional WSN, which performed in-between relaying over Hoyt fading channels. For its comprehensive performance analysis, rapidly converging infinite-series expressions have been provided with focus on the outage probability (OP) and achievable throughput of the hardware-impaired system. Additionally, asymptotic behavior of these performance measures has also been provided. Further, an approach to the symbol-error probability (SEP) analysis is also presented in the context of the observed system. Finally, we consider the shadowing influence along the WSN propagation path. Performance analysis of observed EH system operating over Rician-shadowed fading channels has been carried out, with deriving exact corresponding infinite-series expressions for outage probability (OP) and achievable throughput of the system under the hardware impairment conditions. In addition, bidirectional relaying in a mixed fading environment has been considered.

## 1. Introduction

The future Internet of Things (IoT) tends to connect large number of forthcoming gadgets, and thus wireless sensors are expected to have a crucial role in such interconnected networks. Due to the high volume of implementation of such networks, replacement of the batteries will become a key issue for the massive number of wireless sensors. For effective addressing of underlying sensor’s finite battery problem in IoT, different solutions such as wireless power transfer-powered and energy harvesting-powered wireless sensor networks (WSNs) have emerged as promising remedies [1]. Since WSNs are subjected to limited energy storage at each mobile node, reduction of energy consumption (extension of sensor node battery life) has evolved as a mainstream research issue in WSNs.

Towards this direction, various sources for energy harvesting (EH) have been considered in the literature, i.e., coupled magnetic resonance coils [2], airflow-based [3], solar-based [4], heat-based [5], etc. However, the most sought after technique in this regard is acquiring radio frequency (RF) energy from the surrounding sources. Based on the principle that RF signals can simultaneously transmit energy and information, simultaneous wireless information and power transfer (SWIPT) has emerged as an interesting new area of research for WSNs [1,6].

### State-Of-The-Art

Some of the notable contributions made by the research fraternity in this direction are illustrated as follows.

An EH system overview has been provided in [7], where various EH challenges and considerations involved from architecture, circuits and system perspectives are addressed. In [8], various issues and trade-offs involved in designing and operating of EH embedded systems have been observed. Non-linear EH for a machine-to-machine enabled cellular network has been observed in [9], especially focusing on two different multiple access strategies, namely non-orthogonal multiple access (NOMA) and time division multiple access (TDMA). The total energy minimization problem is formulated as subject to minimal throughput constraints, maximal transmission power constraints and energy causality constraints, with the circuit power consumption taken into account. In addition, possible EH enabling technologies for 6G have been discussed and proposed in [10].

In [11], SWIPT receiver architecture designs based on time switching (TS) and power splitting (PS) have been observed, while the performance analysis of TS EH and PS EH has been carried out in [12]. However, hardware structure of the PS realization is way more complicated than the TS one [13,14,15]. In addition, in reality, not all wireless nodes are equipped with full-duplex capacity due to limitations in hardware or implementation cost [14]. In [15], EH protocol based on TSR architecture for amplify-and-forward (AF) relaying was considered.

Recently, in the literature [16,17,18], special attention has been paid to incorporating the hardware impairment effects into the WSN system performance analysis. Namely, in practice, the transceiver hardware of wireless devices is imperfect because it is affected by impairments such as amplifier-amplitude non-linearity, I/Q imbalance and phase noise [16].

In [19], performance analysis of TS EH was carried out for a case of bidirectional half-duplex (HD) WSN with performed relaying over a Rician fading channel. Performance analysis of EH-based decode-and-forward (DF) relaying in the presence of transceiver imperfections, within Rayleigh fading environment was provided in [20].

The problem of characterization of the random nature of most of the small-scale fading models for WSN communications can be reduced to the problem of characterization of complex Gaussian random process. Hoyt propagation model is used for modeling short-term signal variations arising from the mutual influence of scattered waves. This physical random process can be observed as a complex Gaussian RV with zero mean in-phase and in-quadrature components of different variances [21]. Moreover, this model also serves as a good representation for the scenario for correlated in-phase and in-quadrature components [22]. In addition, it is well-known that this model could be used for modeling propagation environments that are more severe than Rayleigh, and that can be reduced to a special case of Rayleigh model by setting corresponding values of model parameters.

Since Rayleigh model is the widely most used for the transmission particularly when there is no direct line-of-sight (LOS) between the transmitter and the receiver, studying wireless performance measures based on the Hoyt model provides significant generalization of all previous considered scenarios. In [23], authors presented how performance metrics for Hoyt fading can be approximated capitalizing on well-known results for Rayleigh fading. Half-duplex bidirectional WSN with time-switching-based EH relaying protocol over Hoyt fading channels has been observed in [24].

Another contribution of this paper is the consideration of shadowing influence along the WSN propagation path. There arises a need to study the case when obstacles block the LOS link in between network nodes and fluctuations of the LOS signal are brought by shadowing effect. In order to account for the fluctuations of the LOS or scattered signal contributions brought by shadowing effect, several composite fading models have been proposed in the literature [25,26,27]. Here, we will focus on the Rician-shadowed fading model [27], that obtains general properties, since it can cover four different shadowing scenarios: infrequent light shadowing, frequent heavy shadowing, overall shadowing and average shadowing scenario.

In addition, bidirectional relaying in a mixed fading environment will be considered, since observance of mixed fading environments in relaying has become an important topic [28,29,30].

Key contributions of this work are summarized as follows:

- Rapidly converging infinite-series expressions have been derived for the OP and achievable throughput at hardware-impaired destination nodes with information transmission over Rician-shadowed fading environment. Asymptotic analysis and an approximation of the above-mentioned criteria for boundary high SNR values have been delivered when Rician-shadowed fading environment has been observed.
- Rapidly converging infinite-series expressions have been derived for the OP and achievable throughput at hardware-impaired destination nodes with information transmission over the mixed Hoyt/Rician-shadowed fading environment. Asymptotic analysis and an approximation of the above-mentioned criteria for boundary high SNR values have been delivered when the mixed Hoyt/Rician-shadowed fading environment has been observed.
- Rapidly converging infinite-series expressions have been derived for the outage probability (OP) and achievable throughput at hardware-impaired destination nodes with information transmission over Hoyt fading channels. Rapidly converging infinite-series expressions have been derived for the cumulative distribution function (CDF) of the SNR at destination nodes. Further, integral form for the symbol-error probability (SEP) at each node has been presented. Asymptotic analysis and an approximation of the above-mentioned criteria for boundary high SNR values have been delivered.
- Obtained analytical results have been verified through Monte Carlo simulations.

The rest of paper is organized as follows: Section 2 describes the system model and the employed EH protocol. Section 3 and Section 4 provide analysis of system performances along with analysis of their asymptotic values. Further, the obtained numerical results are illustrated in Section 5, while conclusions are drawn in Section 6.

## 2. System Model

**Figure 1:**
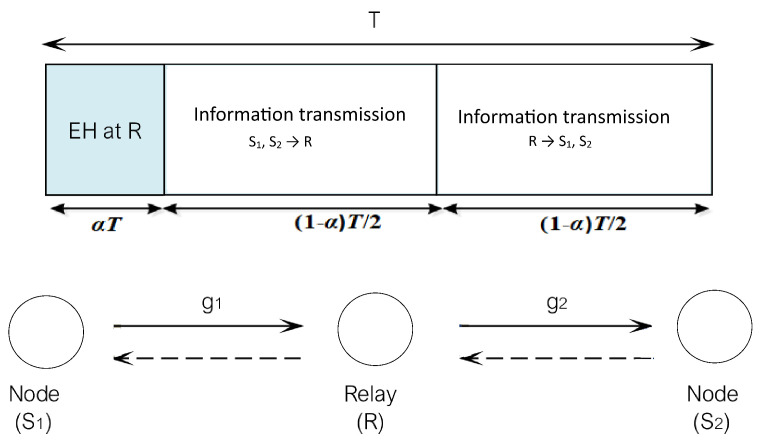
The proposed wireless sensor network (WSN) model with bidirectional relaying, 2019 IEEE [24].

Let us consider a WSN scheme consisting of two sources ($S_{i}$, i=1,2) with bidirectional relaying network over relay node (*R*), as presented at Figure 1. Here, it is assumed that both channel links $S_{1}-R$ and $S_{2}-R$ are exposed to the fading effects, with corresponding channel gains $g_{i}$. It is also assumed that each terminal obtains a single antenna and each terminal operates in HD mode, as explained in [19]. Hardware impairments are here observed at all nodes ($S_{1}$, $S_{2}$ and *R*). Since the direct link between two nodes is not reliable for communication, the communication between sources is assumed to undergo through relay. We are observing the scenario in which relay does not possess sufficient energy both for its own purposes and for forwarding information to system nodes, so EH from source nodes has to be performed before forwarding the information [19]. Additionally, it is also assumed that nodes are aware of the channel gains. Transmission blocks length *T* consists of three time slots of corresponding time lengths: αT, (1−α)T/2 and (1−α)T/2, respectively. As explained in [19], EH from $S_{1}$ and $S_{2}$ is performed at *R* during the first time slot, while at the second time slot, $S_{1}$ and $S_{2}$ simultaneously transmit information to *R*. Finally, after amplifying the signal that he had received, in third time slot *R* broadcasts data to $S_{1}$ and $S_{2}$.

For the case when sensor node $S_{i}$ transmits information symbols $s_{i}$ of average power *P*, $E(|s_{i}{|}^{2})=\;P$, it has been shown in [19] that the SNR value for detection of the symbol $s_{1}$ at $S_{1}$ and $s_{2}$ at $S_{2}$ can be expressed as:

(1) $$\gamma_{1}=\frac{X_{1}X_{2}}{X_{1}X_{2}a+X_{1}^{2}a+X_{1}b+c},$$

and

(2) $$\gamma_{2}=\frac{X_{2}X_{1}}{X_{2}X_{1}a+X_{2}^{2}a+X_{2}b+c},$$

respectively, where $X_{i}={\left|g_{i}\right|}^{2}$ denotes the square of channel gain amplitude on link $S_{i}-R$. Corresponding system parameters are expressed as: $a=\kappa^{2}+\kappa_{r}^{2}\left(1+\kappa^{2}\right)$; $b=\frac{1+\kappa_{r}^{2}}{P/\sigma_{r}^{2}}$; $c=\frac{1+\kappa^{2}}{\psi P/\sigma_{r}^{2}}$; $\psi =\frac{2\eta \alpha }{1-\alpha }$; where η denotes EH efficiency coefficient, $\sigma_{r}^{2}$ denotes the variance of zero-mean additive white Gaussian noise (AWGN) assumed at channels, and parameters κ and $\kappa_{r}$ are used for characterizing the level of hardware impairments in the transmission system [19].

## 3. System Performances

### 3.1. Case of Hoyt Fading Channels

The probability density function (PDF) of Hoyt distributed random process $X_{i}$, *i* = 1, 2, can be expressed as [22]:

(3) $$f_{X_{i}}\left(X_{i}\right)=\frac{1+q_{i}^{2}}{2q_{i}\Omega_{i}}\exp(-\frac{{\left(1-q_{i}^{2}\right)}^{2}X_{i}}{4q_{i}^{2}\Omega_{i}})I_{0}\left(\frac{\left(1-q_{i}^{4}\right)X_{i}}{4q_{i}^{2}\Omega_{i}}\right),$$

where $\Omega_{i}$ denotes the channel average SNR value, $I_{0}\left(x\right)$ is the zero-th order modified Bessel function of the first kind [31], and $0\leq q_{i}\leq 1$ is the desired signal Hoyt fading parameter. The cumulative density function (CDF) of the RV, $X_{i}$ (i=1,2), can be derived as:

(4) $$\begin{array}{c} F_{X_{i}}\left(X_{i}\right)=1-\sum_{k=0}^{\infty }\sum_{m=0}^{2k+1}\frac{{\left(1-q_{i}^{4}\right)}^{2k}q_{i}^{3-2m}X_{i}^{m}\exp\left(-\frac{{\left(1+q_{i}^{2}\right)}^{2}X_{i}}{4q_{i}\Omega_{i}}\right)}{{\left(1+q_{i}^{2}\right)}^{4k-2m+3}m!2^{2k+2m-3}\Omega_{i}^{m}\Gamma \left(k+1\right)k!}. \end{array}$$

Now the OP of this system with respect to a desired threshold, $\gamma_{th}$, can be expressed as [19]:

(5) $$\begin{array}{c} P_{out1}=F_{\gamma_{1}}\left(\gamma_{th}\right)=P_{r}\left(\gamma_{1}<\gamma_{th}\right)=P_{r}\left(\frac{X_{1}X_{2}}{X_{1}X_{2}+aX_{1}^{2}+bX_{1}+c}<\gamma_{th}\right), \end{array}$$

where $\gamma_{th}=2^{2R}-1$, and *R* denotes source transmission rate.

Capitalizing on Equations (3) and (4), as presented in Appendix A, the exact OP for node $S_{1}$ of the proposed system in the presence of Hoyt fading channels can be expressed as:

(6) Pout1=1−∑k=0∞∑m=02k+1∑l=0∞∑r=0m∑p=0rmrrpγth˜mam−rbpk!l!m!Γ(k+1)K2l−2r+p+m+2(2ζ1ζ2)ζ1ζ22l−2r+p+m+22cr−p(1−q24)2k(1−q14)2l(1+q12)exp−(1+q22)2bγth˜4q22Ω2Γ(l+1)22k+2m+6l−3(1+q22)4k−2m+3q22m−3q14l+1Ω12l+2,

where $\zeta_{1}=\frac{{\left(1+q_{2}^{2}\right)}^{2}c\tilde{\gamma_{th}}}{4q_{2}^{2}\Omega_{2}}$, $\zeta_{2}=\frac{{\left(1+q_{2}^{2}\right)}^{2}a\tilde{\gamma_{th}}}{4q_{2}^{2}\Omega_{2}}+\frac{{\left(1+q_{1}^{2}\right)}^{2}}{4q_{1}^{2}\Omega_{1}}$. Similarly to above, the OP at node $S_{2}$ can also be obtained in similar form, by changing corresponding indexes values. Capitalizing on Equation (6), the expression for the achievable throughput of the proposed system in the presence of Hoyt fading channels can be expressed as:

(7) $$\tau_{i}=\left(1-P_{{out}_{i}}\right)\frac{R}{2}\left(1-\alpha \right),\;\;i=1,2.$$

Further capitalizing on $F_{\gamma_{i}}\left(x\right)$ being CDF of $\gamma_{i}$, the SEP at node $S_{i}$ can be expressed as in integral form as:

(8) $$SEP_{i}=\frac{\omega \sqrt{\theta }}{2\sqrt{\pi }}\int_{0}^{\infty }\frac{\exp(-\theta x)}{\sqrt{x}}F_{\gamma_{i}}\left(x\right)dx,$$

where ω and θ are parameters that specify modulation format, as presented in [19].

### 3.2. Case of Rician-Shadowed Fading Channels

The probability density function (PDF) of Rician-shadowed random process $X_{i}$, *i* = 1, 2, can be expressed as [22]:

(9) $$\begin{array}{c} f_{X_{i}}\left(X_{i}\right)={\left(\frac{2b_{i}m_{i}}{2b_{i}m_{i}+\Omega_{i}}\right)}^{m_{i}}\frac{1}{2b_{i}}\exp(-\frac{X_{i}}{2b_{i}}){\;}_{1}F_{1}(m_{i},1,\frac{\Omega_{i}X_{i}}{2b_{i}\left(2b_{i}m_{i}+\Omega_{i}\right)}), \end{array}$$

where ${}_{1}F_{1}\left(x\right)$ denotes the confluent hypergeometric function [31], parameter $2b_{i}$ denotes the average power of scatter component per hop, parameter $\Omega_{i}$ denotes the average power of LOS component per hop, while parameter $m_{i}$ denotes fading severity parameter. It has been shown in [32] that previous SNR PDF expression can be re-written in another form as:

(10) $$\begin{array}{c} f_{X_{i}}\left(X_{i}\right)=\frac{m_{i}^{m_{i}}\left(1+\kappa_{i}\right)}{{\left(\kappa_{i}+m_{i}\right)}^{m_{i}}\bar{x_{i}}}\exp(-\frac{\left(1+\kappa_{i}\right)X_{i}}{\bar{x_{i}}}){\;}_{1}F_{1}(m_{i},1,\frac{\kappa_{i}\left(1+\kappa_{i}\right)X_{i}}{\bar{x_{i}}\left(m_{i}+\kappa_{i}\right)}), \end{array}$$

where parameter $\kappa_{i}$ is defined as ratio of powers, $\kappa_{i}=\frac{\Omega_{i}}{2b_{i}}$, and $\bar{x_{i}}$, $\bar{x_{i}}=E\left[X_{i}\right]$, represents the average channel SNR value. The CDF of the observed SNR $X_{i}$ can be expressed as:

(11) $$\begin{array}{c} F_{X_{i}}\left(X_{i}\right)=1-\sum_{p=0}^{\infty }\sum_{l=0}^{p}\frac{m_{i}\kappa_{i}^{p}{\left(1+\kappa_{i}\right)}^{l}\Gamma \left(m_{i}+p\right)X_{i}^{l}}{{\left(\kappa_{i}+m_{i}\right)}^{m_{i}}{\bar{x_{i}}}^{l}\Gamma \left(m_{i}\right){\left(p!\right)}^{2}l!}\;\exp(-\frac{\left(1+\kappa_{i}\right)X_{i}}{\bar{x_{i}}}). \end{array}$$

Now, after introducing expressions from Equations (10) and (11) into Equation (5), and by following the same procedure presented in Appendix A, the exact OP for node $S_{1}$ of the proposed system in the presence of Rician-shadowed fading channels can be expressed as:

(12) Pout1=1−∑p=0∞∑l=0p∑s=0∞∑q=0l∑w=0qlqqwγth˜lal−qbqκ1sp!l!(s!)2Γ(m1)κ2pcw−q(1+κ1)s+1(1+κ2)lm1m1m2m2Γ(m2)(κ1+m1)m1+s(κ2+m2)m2+px1¯s+1x2¯l×ζ3ζ4l+p−2q+w+12exp−2γth˜(1+κ2)x2¯Γ(m1+s)Γ(m2+p)Kl+p−2q+w+1(2ζ3ζ4),

where $\zeta_{3}=\frac{\left(1+\kappa_{2}\right)c\tilde{\gamma_{th}}}{\bar{x_{2}}}$, $\zeta_{4}=\frac{\left(1+\kappa_{2}\right)\tilde{\gamma_{th}}}{\bar{x_{2}}}a+\frac{\left(1+\kappa_{1}\right)}{\bar{x_{1}}}$.

The OP at node $S_{2}$ can also be obtained in similar form, by changing corresponding indexes values. As in the previous case, having the OP obtained, the achievable throughput can be computed using the Equation (7).

### 3.3. Case of Mixed Hoyt/Rician-Shadowed Fading Channels

It would be also interesting to observe bidirectional relaying scenario when links $S_{1}-R$ and $R-S_{2}$ are exposed to different types of fading. When observing $X_{1}$ to be a Hoyt distributed random process, while $X_{2}$ to be a Rician-shadowed distributed random process, capitalizing on Equations (3), (5) and (11), by following the same procedure as presented in Appendix A, the exact OP for node $S_{1}$ of the proposed relaying system in the presence of a mixed Hoyt/Rician-shadowed fading environment can be expressed as:

(13) Pout1=1−∑k=0∞∑s=0∞∑l=0s∑p=0l∑w=0plppwγth˜lal−kbwcp−wl!k!(s!)2Γ(k+1)m2m2(1+κ2)sκ2s(1−q14)2kΓ(m2+s)q14k+1Γ(m2)(κ2+m2)m2+sx2¯sΩ12k+226k×ζ5ζ62k+l+w−2p+22exp−γthb˜(1+κ2)x2¯K2k+l+w−2p−2(2ζ5ζ6),

where $\zeta_{5}=\frac{\left(1+\kappa_{2}\right)\tilde{\gamma_{th}}}{c\bar{x_{2}}}$, $\zeta_{6}=\frac{\left(1+\kappa_{2}\right)\tilde{\gamma_{th}}}{\bar{x_{2}}}a+\frac{{\left(1+q_{1}^{2}\right)}^{2}}{4q_{1}^{2}\Omega_{1}}$.

Similarly, capitalizing on Equations (4), (5) and (10), by following the same procedure as presented in Appendix A, the exact OP for node $S_{2}$ of the proposed system in the presence of a mixed Hoyt/Rician-shadowed fading environment can be expressed as:

(14) Pout2=1−∑k=0∞∑s=02k+1∑p=0∞∑r=0s∑l=0rsrrlγth˜sas−rblcr−ls!k!(p!)2Γ(k+1)m1m1(1+κ1)p+1(1−q24)2kΓ(m1+p)q22s−3Γ(m1)(κ1+m1)m1+px1¯p+1Ω2s×ζ7ζ8s−r+22Ks−r+2(2ζ7ζ8)22k+2s−4(1+q22)4k−2s+3,

where $\zeta_{7}=\frac{{\left(1+q_{2}^{2}\right)}^{2}c\tilde{\gamma_{th}}}{4q_{2}^{2}\Omega_{2}}$, $\zeta_{8}=\frac{{\left(1+q_{2}^{2}\right)}^{2}\tilde{\gamma_{th}}}{4q_{2}^{2}\Omega_{2}}a+\frac{\left(1+\kappa_{1}\right)}{\bar{x_{1}}}$.

It is here important to notice that special respect should be paid to the task of determining the optimal ratio between the time allocated for EH and the time allocated for information transmission. Determination of an optimal protocol for TS would provide the best throughput performance, in a way that as higher as possible available transmission power would be obtained, while keeping at the same time the transmission rate as high as possible. An efficient approach for performing such a task would be solving $\frac{d\tau_{i}\left(\alpha \right)}{d\alpha }=0$. However, Equations (7) and (12)–(14) are written in the form of infinite series and a modified Bessel function of second kind, and it is hard to provide a closed-form solution for this case. An alternative approach is obtaining this solution numerically. In the literature, various algorithms for such optimization have been presented [33,34].

## 4. Asymptotic Analysis

### 4.1. Case of Hoyt Fading Channels

In this subsection, asymptotic analysis for the high SNR regime will be carried out. In such a way, correctness of the exact analysis will be verified. As the $P/N_{0}$ obtains very high values, the SNR in Equations (1) and (2) asymptotically tends to:

(15) $$\gamma_{1}^{\infty }=\frac{X_{2}}{a(X_{1}+X_{2})},$$

and

(16) $$\gamma_{2}^{\infty }=\frac{X_{1}}{a(X_{1}+X_{2})}.$$

It has been shown in [19] that asymptotic OP at node $S_{1}$ can be expressed as:

(17) $$\begin{array}{ccc} & & P_{out_{1}}^{\infty }=Pr\left(\gamma_{1}^{\infty }<\gamma_{th}\right)=Pr\left(\frac{X_{1}}{X_{1}+X_{2}}<a\gamma_{th}\right)=\int_{0}^{\infty }F_{X_{2}}\left(\frac{a\gamma_{th}x_{1}}{1-a\gamma_{th}}\right)f_{X_{1}}\left(x_{1}\right)dx_{1}. \end{array}$$

Capitalizing on Equations (3) and (4), as presented in Appendix B, the asymptotic OP for node $S_{1}$ of the proposed system in the presence of Hoyt fading channels can be expressed as:

(18) $$\begin{array}{c} P_{{out}_{1}}=1-\sum_{k=0}^{\infty }\sum_{l=0}^{\infty }\sum_{m=0}^{2k+1}\frac{{\left(1-q_{2}^{4}\right)}^{2k}{\left(1-q_{1}^{4}\right)}^{2l}{\left(1-q_{1}\right)}^{2}}{k!l!m!\Gamma \left(k+1\right)\Gamma \left(l+1\right)q_{2}^{2m-3}}\frac{\Gamma \left(m+2l+2\right){\left(\frac{a\gamma_{th}}{1-a\gamma_{th}}\right)}^{m}\xi_{2}^{-(m+2l+2)}}{{\left(1+q_{2}^{2}\right)}^{4k-2m+3}2^{2k+2m+6l-3}\Omega_{2}^{m}\Omega_{1}^{2l+2}q_{1}^{4l+1}}. \end{array}$$

OP at $S_{2}$ can be expressed by exchanging indices in Equation (18).

### 4.2. Case of Rician-Shadowed Fading Channels

Starting from Equations (10) and (11), asymptotic OP for node $S_{1}$ of the proposed system in the presence of Rician-shadowed fading channels can be expressed as:

(19) $$\begin{array}{ccc} P_{{out}_{1}} & = & 1-\sum_{p=0}^{\infty }\sum_{s=0}^{\infty }\sum_{l=0}^{s}\frac{{\left(1+\kappa_{1}\right)}^{p+1}{\left(1+\kappa_{2}\right)}^{s}\kappa_{1}^{p}\kappa_{2}^{s}m_{1}^{m_{1}}m_{2}^{m_{2}}}{{\left(p!\right)}^{2}l!{\left(s!\right)}^{2}\Gamma \left(m_{1}\right)\Gamma \left(m_{2}\right){\bar{x_{1}}}^{p+1}{\bar{x_{2}}}^{s}}\\ & & \times \frac{\Gamma \left(p+l+1\right)\Gamma \left(m_{1}+p\right)\Gamma \left(m_{2}+l\right){\left(\frac{a\gamma_{th}}{1-a\gamma_{th}}\right)}^{l}}{{\left(\kappa_{1}+m_{1}\right)}^{m_{1}+p}{\left(\kappa_{2}+m_{2}\right)}^{m_{2}+s}{\left(\frac{a\gamma_{th}\left(1+\kappa_{2}\right)}{1-a\gamma_{th}\bar{x_{2}}}+\frac{1+\kappa_{1}}{\bar{x_{1}}}\right)}^{p+l+1}}. \end{array}$$

OP at $S_{2}$ can be expressed by exchanging indices in Equation (19).

### 4.3. Case of Mixed Hoyt/Rician-Shadowed Fading Channels

Starting from Equations (3) and (11), by following the same procedure as presented in Appendix B, asymptotic OP for node $S_{1}$ of the proposed system in the presence of a mixed Hoyt/Rician-shadowed fading environment can be expressed as:

(20) $$\begin{array}{ccc} P_{{out}_{1}} & = & 1-\sum_{s=0}^{\infty }\sum_{l=0}^{s}\sum_{p=0}^{\infty }\frac{{\left(1+\kappa_{2}\right)}^{s}\kappa_{2}^{s}m_{2}^{m_{2}}\left(1+q_{1}^{2}\right){\left(1-q_{1}^{4}\right)}^{2p}}{{\left(s!\right)}^{2}l!p!\Gamma \left(m_{2}\right){\bar{x_{2}}}^{s}\Gamma \left(p+1\right)\Omega_{1}^{2p+2}2^{6p+1}}\\ & & \times \frac{\Gamma \left(m_{2}+s\right)\Gamma \left(2p+l+2\right){\left(\frac{a\gamma_{th}}{1-a\gamma_{th}}\right)}^{l}}{{\left(\kappa_{2}+m_{2}\right)}^{m_{2}+s}q_{1}^{4p+1}{\left(\frac{{\left(1+q_{1}^{2}\right)}^{2}}{4q_{1}^{2}\Omega_{1}}+\frac{a\gamma_{th}\left(1+\kappa_{2}\right)}{\left(1-a\gamma_{th}\right)\bar{x_{2}}}\right)}^{2p+l+2}}. \end{array}$$

Similarly, starting from Equations (4) and (10), by following the same procedure as presented in Appendix B, asymptotic OP for node $S_{2}$ of the proposed system in the presence of a mixed Hoyt/Rician-shadowed fading environment can be expressed as:

(21) $$\begin{array}{ccc} P_{{out}_{2}} & = & 1-\sum_{k=0}^{\infty }\sum_{l=0}^{2k+1}\sum_{p=0}^{\infty }\frac{{\left(1+\kappa_{1}\right)}^{p+1}\kappa_{1}^{p}m_{1}^{m_{1}}{\left(1-q_{2}^{4}\right)}^{2k}}{{\left(p!\right)}^{2}k!l!\Gamma \left(m_{1}\right)\Gamma \left(k+1\right){\bar{x_{1}}}^{p+1}\Omega_{2}^{l}q_{2}^{2l-3}2^{2k+2l-3}}\\ & & \times \frac{\Gamma \left(p+l+1\right)\Gamma \left(m_{1}+p\right){\left(\frac{a\gamma_{th}}{1-a\gamma_{th}}\right)}^{l}}{{\left(\kappa_{1}+m_{1}\right)}^{m_{1}+p}{\left(1+q_{2}^{2}\right)}^{4k-2l+3}{\left(\frac{a\gamma_{th}{\left(1+q_{2}^{2}\right)}^{2}}{\left(1-a\gamma_{th}\right)4q_{2}^{2}\Omega_{2}}+\frac{1+\kappa_{1}}{\bar{x_{1}}}\right)}^{p+l+1}}. \end{array}$$

## 5. Numerical Results

First we will observe the Hoyt fading channels case. In order to validate the correctness of the derived rapidly converging infinite-series OP expressions, Monte Carlo simulations have been carried out. The parameters related to the impairments of hardware will be set as κ = $\kappa_{r}$ = 0.1, while a case without impairment (κ = $\kappa_{r}$ = 0) is also observed. The Hoyt channel properties are considered with $\Omega_{1}=\Omega_{2}$ = 0.5 and with the Hoyt *q* fading severity parameters spanning from 0.5 to 0.8 for both channels. All the other observed parameter values for the EH efficiency, source transmission rate and source SNR are provided in Table 1.

**Figure 2:**
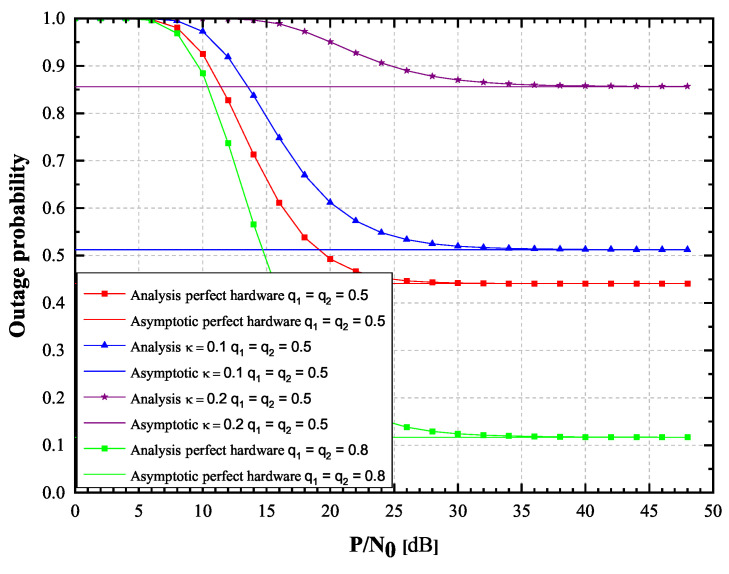
Hoyt fading channels: Outage probability versus $P/N_{0}$, 2019 IEEE [24].

**Figure 3:**
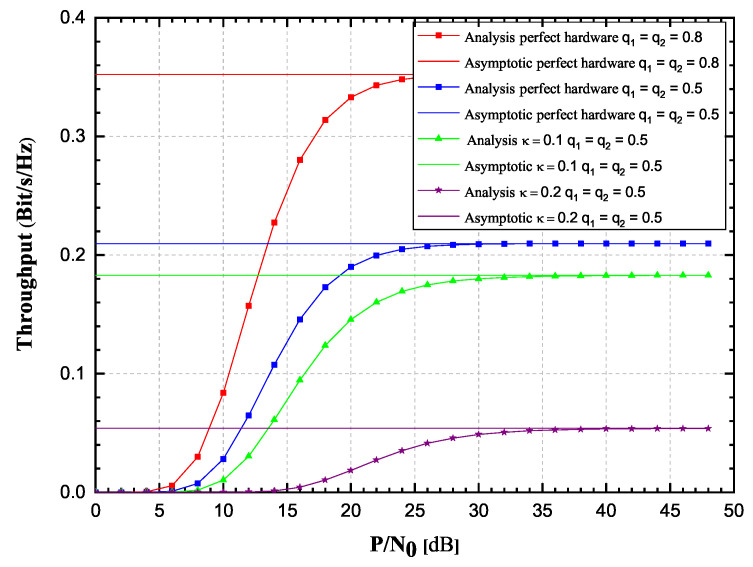
Hoyt fading channels: Achievable throughput versus $P/N_{0}$, 2019 IEEE [24].

In Figure 2 and Figure 3, OP and achievable throughput of the observed system are presented in the function of source SNR. A case of α = 0.5 has been considered, when the EH time and the duration of information transmission are equal. It can be seen from the figures that OP and throughput values obtained by using derived infinite-series expressions and their corresponding values obtained by Monte Carlo simulations excellently concede. From Figure 2 and Figure 3, it is clearly visible that OP values increase, while throughput values decrease as κ varies from 0 to 0.2. Additionally, it is also visible that for high SNR values, the OP and throughput values tend to approach the corresponding asymptotic values, in a manner that the lower the value of κ, the faster the asymptotic values will be reached. Figures also depict how change in fading severity (*q* parameter values change from 0.8 to 0.5) significantly deteriorates system performances. Perfect hardware feature corresponds to the practical case when hardware is novel or repaired.

**Figure 4:**
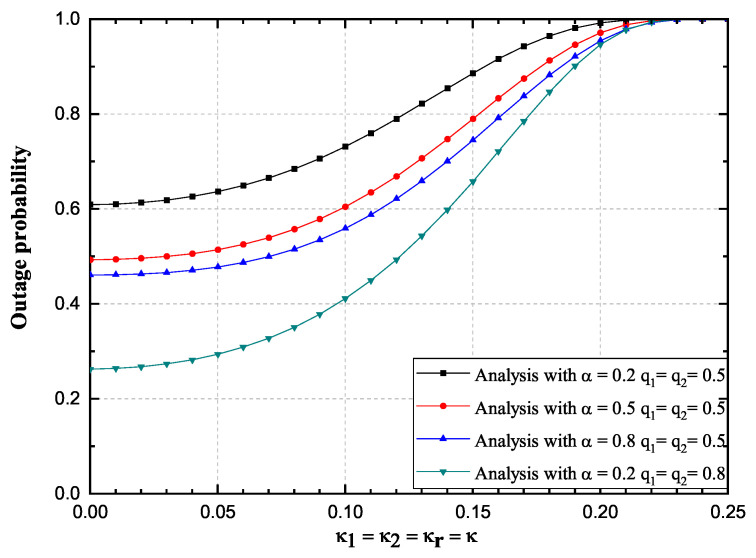
Hoyt fading channels: Outage probability versus κ, 2019 IEEE [24].

**Figure 5:**
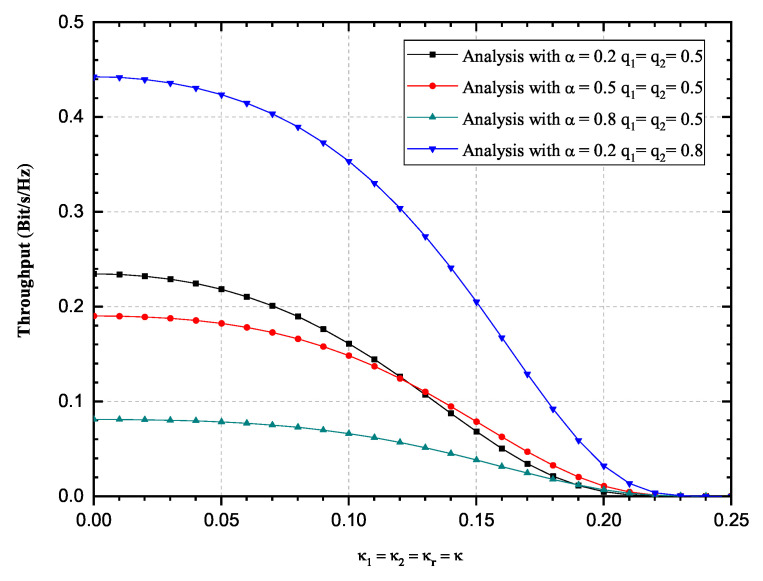
Hoyt fading channels: Achievable throughput versus κ, 2019 IEEE [24].

Influence of impaired hardware on the OP and the achievable throughput is observed more closely in Figure 4 and Figure 5, for a single SNR value of 20 dB and fixed transmission rate value. Three scenarios have been observed, i.e., α = 0.2, 0.5, 0.8. Again, it is validated that values obtained by using derived infinite-series expressions excellently match with the corresponding values obtained by Monte Carlo simulations. From Figure 4 and Figure 5, it is obvious that the OP values increase significantly while the achievable throughput values decrease with the increase of the impairment level κ. The influence of parameter α values change on the OP values decrease is non-negligible, since a higher amount of power is used for information transmission. The influence of parameter α values change on the achievable throughput values is more complex, since it can be seen from Figure 5, that the throughput performance is improved when α increases from 0.2 to 0.5, while throughput performance deteriorates when α increases from 0.5 to 0.8. Significant influence of fading severity change is visible from the figures.

Let us now observe the Rician-shadowed fading channels case. Rician-shadowed link parameter values for the corresponding shadowing mode are provided in [27] as: heavy shadowing ($m_{1}$ = $m_{2}$ = 0.739, $b_{1}$ = $b_{2}$ = 0.063, $\kappa_{1}$ = $\kappa_{2}$ = 0.00711, $\bar{x_{1}}$ = $\bar{x_{2}}$ = 8.97 $\times \;10^{-4}$) average shadowing ($m_{1}$ = $m_{2}$ = 10.1, $b_{1}$ = $b_{2}$ = 0.126, $\kappa_{1}$ = $\kappa_{2}$ = 4.0828, $\bar{x_{1}}$ = $\bar{x_{2}}$ = 0.835), overall shadowing ($m_{1}$ = $m_{2}$ = 5.21, $b_{1}$ = $b_{2}$ = 0.251, $\kappa_{1}$ = $\kappa_{2}$ = 0.55387, $\bar{x_{1}}$ = $\bar{x_{2}}$ = 0.278) and light shadowing ($m_{1}$ = $m_{2}$ = 19.4, $b_{1}$ = $b_{2}$ = 0.158, $\kappa_{1}$ = $\kappa_{2}$ = 2.64241, $\bar{x_{1}}$ = $\bar{x_{2}}$ = 1.29). Hardware impairment parameter values and the other observed parameter values for the EH efficiency, source transmission rate and source SNR will be the same as for the Hoyt fading channels case.

**Figure 6:**
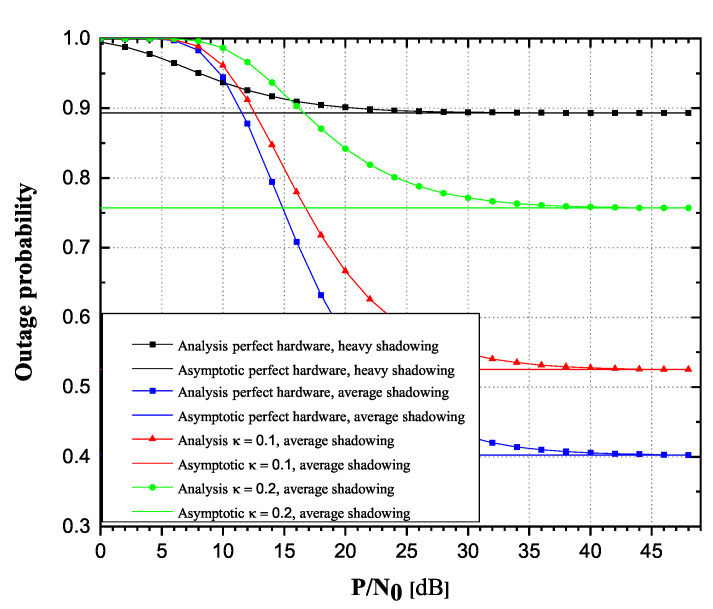
Rician-shadowed fading channels: Outage probability versus $P/N_{0}$.

**Figure 7:**
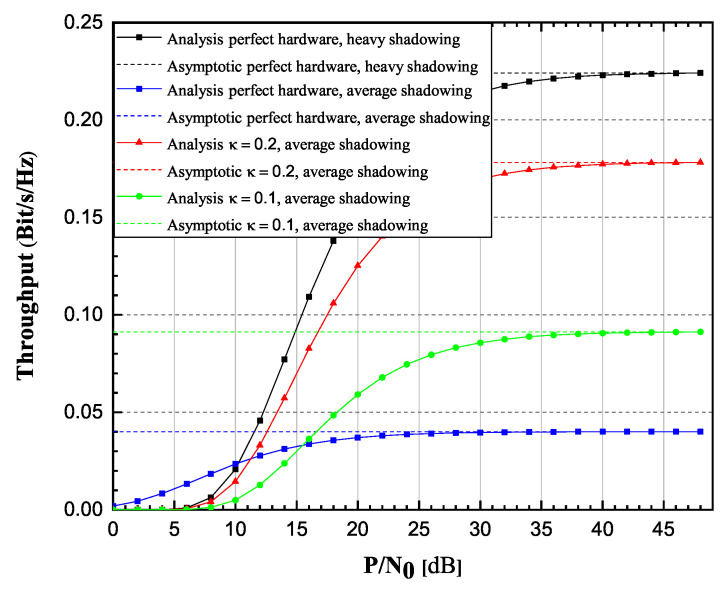
Rician-shadowed fading channels: Achievable throughput versus $P/N_{0}$.

From Figure 6 and Figure 7, it is clearly visible that OP values increase, while throughput values decrease as κ varies from 0 to 0.2. Additionally, it is also visible that for high SNR values, the OP and throughput values tend to approach the corresponding values obtained from asymptotic expressions, with a tendency of reaching asymptotic values more prompt for lower values of κ parameter. Figures also depict how strong the influence of shadowing severity over the links could be. It is clearly visible how heavy shadowing significantly deteriorates system performances, compared to the case of average shadowing.

**Figure 8:**
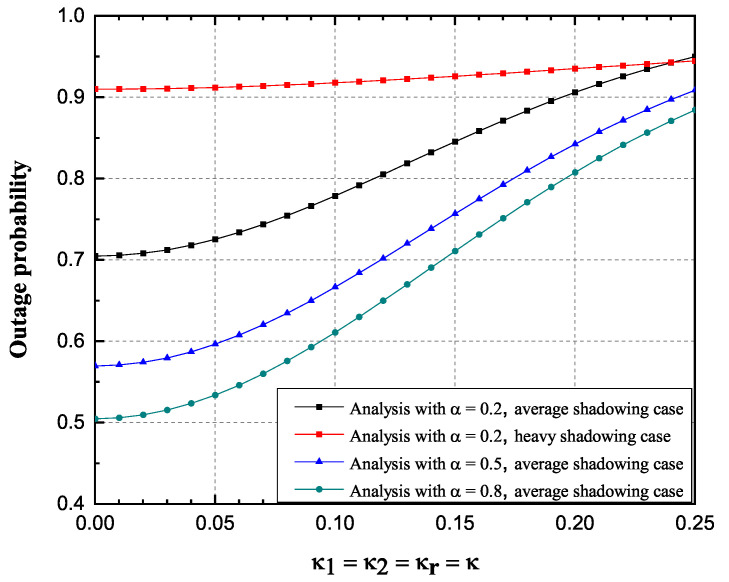
Rician-shadowed fading channels: Outage probability versus κ.

**Figure 9:**
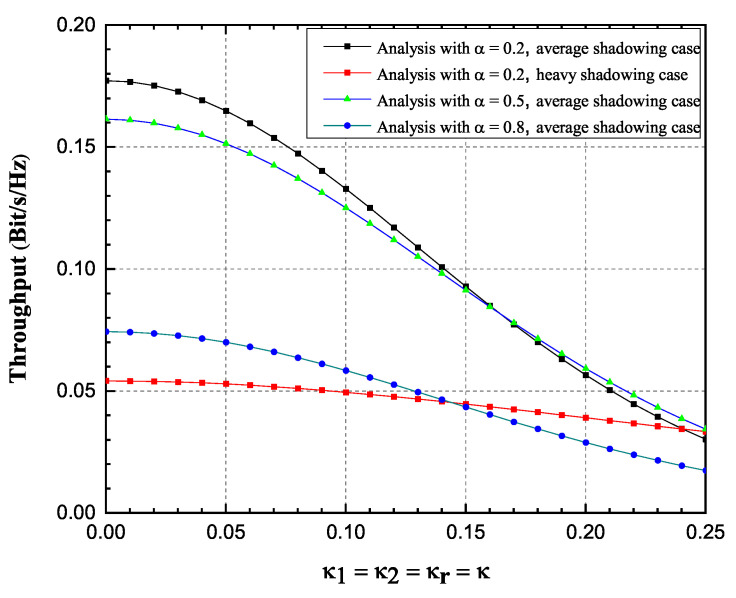
Rician-shadowed fading channels: Achievable throughput versus κ.

Influence of impaired hardware on the OP and the achievable throughput is observed more closely in Figure 8 and Figure 9. Three scenarios have been observed, α = 0.2, 0.5, 0.8.

From Figure 8 and Figure 9, it is obvious that the achievable throughput values decrease while the OP values notably increase with the increase of the impairment level κ. In addition, OP reduces for higher values of TS factor, since higher amount of power is used for data transmission. Significant influence of shadowing severity values change on the achievable throughput values is also visible.

**Figure 10:**
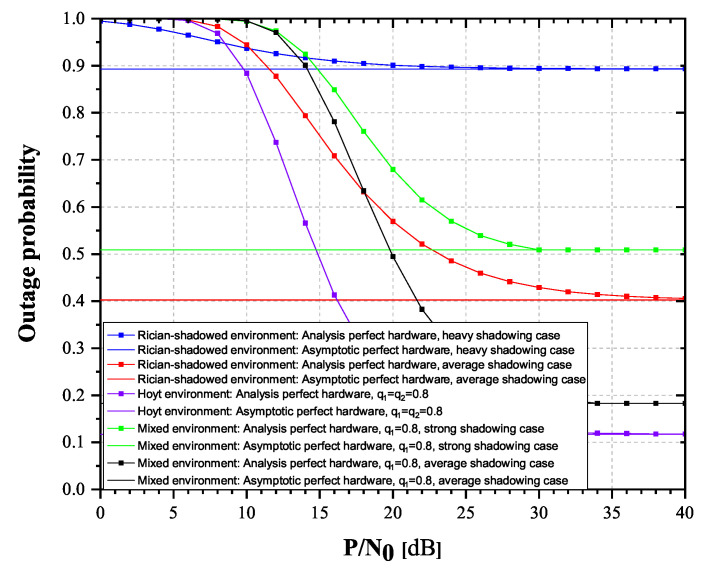
Influence of fading environment: Outage probability versus $P/N_{0}$.

**Figure 11:**
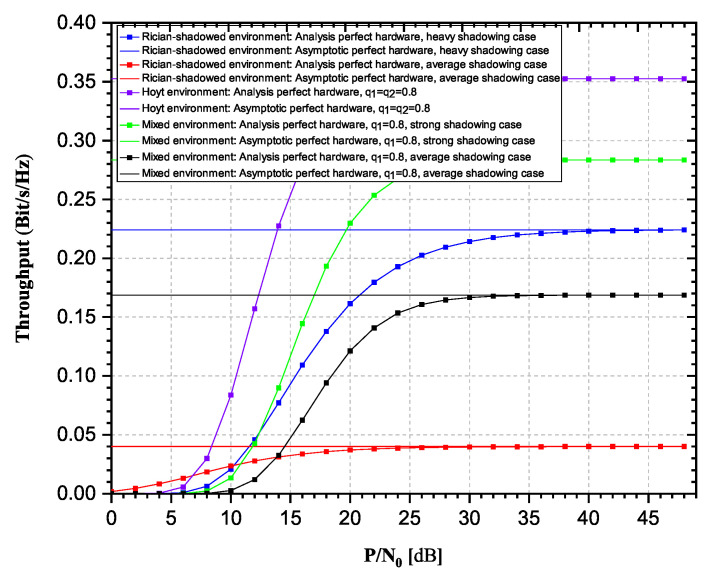
Influence of fading environment: Achievable throughput versus $P/N_{0}$.

Finally we can observe relaying performances of system operating over mixed Hoyt/Rician-shadowed fading channels. In Figure 10 and Figure 11, it is visible that the influence of Hoyt fading experienced at one hop determines the behavior of system performances more significantly than the influence of Rician-shadowed fading experienced at the other hop. This can be explained by the more severe nature of Hoyt fading channels compared to severity of Rician-shadowed fading.

## 6. Conclusions

In this work, a detailed performance analysis of an HD bidirectional WSN over a cooperative relay system is presented. Relaying is TS-based with EH over Hoyt fading channels. In addition to considering a general Hoyt propagation scenario, the work also considered effects of hardware impairments at cooperative nodes. Further, rapidly converging infinite-series expressions for the OP and achievable throughput have been derived, along with provided asymptotic analysis, and verified by using Monte Carlo simulations. System performances have been observed and discussed in the function of various WSN system parameters. Another contribution of this paper is consideration of shadowing influence along the WSN propagation path. The performance analysis of the observed EH system, operating over Rician-shadowed fading channels, has been carried out by deriving exact corresponding infinite-series expressions, asymptotic analysis and providing corresponding discussion about the effects of various parameters on performances of observed WSN system. Finally, we have observed a hypothetical case of relaying over mixed Hoyt/Rician-shadowed fading channels. For such a scenario, rapidly converging infinite-series expressions for the OP and achievable throughput have been derived, along with the provided asymptotic analysis. For such a case, system performances have been also observed and discussed in the function of various WSN system parameters.

## Figures and Tables

**Table 1:** Observed values of system parameters, 2019 IEEE [24].

Symbol	Parameter Names	Values
λ	Energy harvesting efficiency	0.7
$\Omega_{1}$	Mean of $g_{1}^{2}$	0.5
$\Omega_{2}$	Mean of $g_{2}^{2}$	0.5
$q_{1}=q_{2}$	fading severity parameter	0.5, 0.8
$P/N_{0}$	Source SNR	0–50 dB
$\kappa =\kappa_{r}$	Hardware impairment parameter	0, 0.1, 0.2
R	Transmission rate at source	1.5 bps/Hz

## Appendix A

As stated, the exact OP for node $S_{1}$ of the proposed system in the presence of Hoyt fading channels can be calculated according to:

(A1) $$\int_{0}^{\infty }F_{X_{2}}\left(\frac{\gamma_{th}x_{1}^{2}a+\gamma_{th}x_{1}b+\gamma_{th}c}{x_{1}-\gamma_{th}x_{1}a}\right)f_{x_{1}}\left(x_{1}\right)dx.$$

Let us express $\tilde{\gamma_{th}}=\frac{\gamma_{th}}{1-a\gamma_{th}}$, then $\frac{\gamma_{th}x_{1}^{2}a+\gamma_{th}x_{1}b+\gamma_{th}c}{x_{1}-\gamma_{th}x_{1}a}$ transforms to $\tilde{\gamma_{th}}\left(x_{1}a+\frac{c}{x_{1}}+b\right)$. By using the well-known binomial expression ${\left(x+y\right)}^{m}=\sum_{n=0}^{m}$mn$x^{m-n}y^{n}$, along with Equation (3.471.9) from [31], and by introducing $\zeta_{1}=\frac{{\left(1+q_{2}^{2}\right)}^{2}c\tilde{\gamma_{th}}}{4q_{2}^{2}\Omega_{2}}$, $\zeta_{2}=\frac{{\left(1+q_{2}^{2}\right)}^{2}a\tilde{\gamma_{th}}}{4q_{2}^{2}\Omega_{2}}+\frac{{\left(1+q_{1}^{2}\right)}^{2}}{4q_{1}^{2}\Omega_{1}}$, we obtain:

Pout1=1−∑k=0∞∑m=02k+1∑l=0∞∑r=0m∑p=0rmrrpγth˜mam−rbpk!l!m!Γ(k+1)ζ1ζ22l−2r+p+m+22K2l−2r+p+m+2(2ζ1ζ2)cr−p(1−q24)2k(1−q14)2l(1+q12)exp−(1+q22)2bγth˜4q22Ω2Γ(l+1)22k+2m+6l−3(1+q22)4k−2m+3q22m−3q14l+1Ω12l+2,

where $K_{v}\left(x\right)$ is the modified Bessel function of the second kind of order *v*.

## Appendix B

The asymptotic OP at node $S_{1}$ can be expressed as:

$$P_{out_{1}}^{\infty }=Pr\left(\gamma_{1}^{\infty }<\gamma_{th}\right)=Pr\left(\frac{X_{1}}{X_{1}+X_{2}}<a\gamma_{th}\right)=\int_{0}^{\infty }F_{X_{2}}\left(\frac{a\gamma_{th}x_{1}}{1-a\gamma_{th}}\right)f_{X_{1}}\left(x_{1}\right)dx_{1}.$$

Now by using (3.381.4) from [31], we derive the asymptotic expression in the form of:

$$P_{{out}_{1}}=1-\sum_{k=0}^{\infty }\sum_{l=0}^{\infty }\sum_{m=0}^{2k+1}\frac{{\left(1-q_{2}^{4}\right)}^{2k}{\left(1-q_{1}^{4}\right)}^{2l}{\left(1-q_{1}\right)}^{2}}{k!l!m!\Gamma \left(k+1\right)\Gamma \left(l+1\right)q_{2}^{2m-3}}\frac{\Gamma \left(m+2l+2\right){\left(\frac{a\gamma_{th}}{1-a\gamma_{th}}\right)}^{m}\xi_{2}^{-(m+2l+2)}}{{\left(1+q_{2}^{2}\right)}^{4k-2m+3}2^{2k+2m+6l-3}\Omega_{2}^{m}\Omega_{1}^{2l+2}q_{1}^{4l+1}}.$$

## References

[1] Jayakody D.N.K., Thompson J., Chatzinotas S., Durrani S. (2017). Wireless Information and Power Transfer: A New Paradigm for Green Communications.

[2] Guo S., Wang C., Yang Y. (2014). Joint mobile data gathering and energy provisioning in wireless rechargeable sensor networks. IEEE Trans. Mob. Comput..

[3] Kosunalp S. (2017). An energy prediction algorithm for wind-powered wireless sensor networks with energy harvesting. Energy.

[4] Wang C., Li J., Yang Y., Ye F. (2017). Combining solar energy harvesting with wireless charging for hybrid wireless sensor networks. IEEE Trans. Mob. Comput..

[5] Prijic A., Vracar L., Vuckovic D., Milic D., Prijic Z. (2015). Thermal energy harvesting wireless sensor node in aluminum core PCB technology. IEEE Sens. J..

[6] Varshney L.R. Transporting information and energy simultaneously. Proceedings of the 2008 IEEE International Symposium on Information Theory.

[7] Lu C., Raghunathan V., Roy K. (2011). Efficient Design of Micro-Scale Energy Harvesting Systems. IEEE J. Emerg. Sel. Top. Circuits Syst..

[8] Raghunathan V., Chou P. Design and power management of energy harvesting embedded systems. Proceedings of the 2006 International Symposium on Low Power Electronics and Design.

[9] Yang Z., Xu W., Pan Y., Pan C., Chen M. (2018). Energy efficient resource allocation in machine-to-machine communications with multiple access and energy harvesting for IoT. IEEE Internet Things J..

[10] Saad W., Bennis M., Chen M. (2020). A vision of 6G wireless systems: Applications trends technologies and open research problems. IEEE Netw..

[11] Zhou X., Zhang R., Ho C.K. (2013). Wireless information and power transfer: Architecture design and rate-energy trade off. IEEE Trans. Commun..

[12] Nasir A.A., Zhou X., Durrani S., Kennedy R.A. (2013). Relaying protocols for wireless energy harvesting and information processing. IEEE Trans. Wirel. Commun..

[13] Do D.T., Van Nguyen M.S., Hoang T.A., Voznak M. (2019). NOMA-Assisted Multiple Access Scheme for IoT Deployment: Relay Selection Model and Secrecy Performance Improvement. Sensors.

[14] Phan V.D., Nguyen T.N., Tran M., Trang T.T., Voznak M., Ha D.H., Nguyen T.L. (2019). Power Beacon-Assisted Energy Harvesting in a Half-Duplex Communication Network under Co-Channel Interference over a Rayleigh Fading Environment: Energy Efficiency and Outage Probability Analysis. Energies.

[15] Do D.T., Le C.B. (2018). Application of NOMA in Wireless System with Wireless Power Transfer Scheme: Outage and Ergodic Capacity Performance Analysis. Sensors.

[16] Bjornson E., Matthaiou M., Debbah M. (2013). A New Look at Dual-Hop Relaying: Performance Limits with Hardware Impairments. IEEE Trans. Commun..

[17] Nguyen T.N., Duy T.T., Luu G.T., Tran P.T., Voznak M. (2017). Energy harvesting-based spectrum access with incremental cooperation, relay selection and hardware noises. Radioengineering.

[18] Peng C., Li F., Liu H. (2017). Wireless energy harvesting two-way relay networks with hardware impairments. Sensors.

[19] Nguyen T., Minh T.Q., Tran P., Voznak M. (2018). Energy harvesting over Rician fading channel: A performance analysis for half-duplex bidirectional sensor networks under hardware impairments. Sensors.

[20] Nguyen D.K., Jayakody D.N.K., Chatzinotas S., Thompson J.S., Li J. (2017). Wireless energy harvesting assisted two-way cognitive relaynetworks: Protocol design and performance analysis. IEEE Access.

[21] Simon M., Alouini M. (2001). Digital Communication over Fading Channels.

[22] Panic S., Stefanovic M., Anastasov J., Spalevic P. (2013). Fading and Interference Mitigation in Wireless Communications.

[23] Romero-Jerez J.M., Lopez-Martinez F.J. (2017). A new frameworkfor the performance analysis of wireless communications under hoyt (Nakagami-q) Fading. IEEE Trans. Inf. Theory.

[24] Panic S., Jayakody D.N.K., Garg S. Self-Energized Bidirectional Sensor Networks over Hoyt Fading Channels under Hardware Impairments. Proceedings of the 2019 IEEE 90th Vehicular Technology Conference (VTC2019-Fall).

[25] Yoo S.K., Bhargav N., Cotton S.L., Sofotasios P.C., Matthaiou M., Valkama M., Karagiannidis G.K. (2017). The *κ*-*μ*/inverse gamma and *η*-*μ*/inverse gamma composite fading models. IEEE Trans. Inf. Theory.

[26] Stamenovic G., Panic S., Rancic D., Stefanovic C., Stefanovic M. (2014). Performance analysis of wireless communication system in general fading environment subjected to shadowing and interference. EURASIP J. Wirel. Commun. Netw..

[27] Abdi A., Lau W., Alouini M.S., Kaveh M. (2003). A new simple modelfor land mobile satellite channels: First- and second-order statistics. IEEE Trans. Wirel. Commun..

[28] Pandey A., Yadav S. (2018). Physical layer security in cooperative AF relaying networks with direct links over mixed Rayleigh and double-Rayleigh fading channels. IEEE Trans. Veh. Technol..

[29] Pandey A., Yadav S. (2019). Physical layer security in cooperative amplify-and-forward relay networks over mixed Nakagami-m and double Nakagami-m fading channels: Performance evaluation and optimisation. IET Commun..

[30] Raza W., Nasir H., Javaid N. (2020). Unification of RF energy harvesting schemes under mixed Rayleigh-Rician fading channels. AEU-Int. J. Electron. Commun..

[31] Zwillinger D., Moll V., Gradshteyn I.S., Ryzhik I.M. (2015). Table Ofintegrals, Series, and Products.

[32] Bhargav N., Silva R.D., Cotton S., Sofotasios P., Yacoub M.D. (2018). Double Shadowing the Rician Fading Model. IEEE Wirel. Commun. Lett..

[33] Duong T.Q., Duy T.T., Matthaiou M., Tsiftsis T., Karagiannidis G.K. Cognitive cooperative networks in dual-hop asymmetric fading channels. Proceedings of the 2013 IEEE Global Communications Conference (GLOBECOM).

[34] Chong E.K., Zak S.H. (2013). An Introduction to Optimization.

